# Burnout among Swedish school teachers – a cross-sectional analysis

**DOI:** 10.1186/s12889-016-3498-7

**Published:** 2016-08-18

**Authors:** Inger Arvidsson, Carita Håkansson, Björn Karlson, Jonas Björk, Roger Persson

**Affiliations:** 1 Occupational and Environmental Medicine, Lund University, SE-221 85 Lund, Sweden; 2 Department of Psychology, Lund University, Lund, Sweden; 3 Centre for Medicine and Technology for Working Life and Society (Metalund), Lund, Sweden

**Keywords:** Exhaustion, Leisure, Psychosocial working conditions, Stress, Work

## Abstract

**Background:**

Teachers are at high risk of stress-related disorders. This study aimed to examine the occurrence of burnout in a sample of Swedish school-teachers, to test a combined measure of three burnout dimensions on the individual level, to characterize associations between burnout and factors encountered during work and leisure time, and to explore any differences between the genders.

**Methods:**

A questionnaire of occupational, sociodemographic and life-style factors was answered by 490 teachers in school years 4–9. Outcome measures were (a) the single burnout dimensions of exhaustion, cynicism and professional efficacy (Maslach Burnout Inventory-General Survey), and (b) a combined measure based on high or low values in the three dimensions. The combined measure was used to stratify the study population into four levels (0–3) of burnout. Multivariable Poisson regression was applied on level 2 + 3 vs. level 0 + 1, for variables that we considered as relevant risk factors for burn out.

**Results:**

Half of the teachers reported low values in all three dimensions (level 0), whereas 15 were classified as having high burnout in at least two out of the three dimensions (level 2 + 3), and 4 % in all three dimensions (level 3). Almost all psychosocial factors were incrementally more unfavourably reported through the rising levels of burnout, and so were dissatisfaction with the computer workstation, pain, sleep problems and lack of personal recovery. There was no association between gender and rising levels of overall burnout (*p* > 0.30). Low self-efficacy, poor leadership, high job demands and teaching in higher grades were the variables most clearly associated with burnout in multivariable Poisson regression.

**Conclusions:**

Even if circa 50 % of the teachers appear do well with respect to burnout, the results points to the need of implementing multifaceted countermeasures that may serve to reduce burnout.

## Background

The stressful working conditions of teachers have attracted researcher’s interest in many countries [1–5]. One stress-related consequence teachers may experience is burnout, which is an undesirable psychological state characterized by exhaustion, cynicism and feelings of reduced professional efficacy [6]. The proposed explanations for teacher burnout are many and include associations between burnout and a strained work situation (i.e., high demands in combination with low job control), limited resources, permeable boundaries between private life and individual characteristics [7–10].

In Sweden, recent official statistics indicate that teachers are among the occupational groups with the highest risk of stress-related disorders [11, 12]. However, while several previous studies in an international context illuminate the extent of which school-teachers experience burnout [1–5, 8], such research concerning Swedish school-teachers is lacking. Yet, the teachers in Sweden has during the recent decades been subjected to many and wide ranging school reforms, including organizational changes, new legislation and governance of schools, and an extensive privatization. In fact, the Swedish system allows private schools to take out large profit-margins, which is unique from an international perspective. In any event, these changes and reforms have created a competitive and unpredictable situation that influences the work environments in both public and private schools. Acknowledging this, as well as the differences in legislations, educational systems and culture [13] which complicates the translation of results between countries, there is a need to investigate the occurrence of burnout among Swedish school-teachers.

Using the Maslach Burnout Inventory-General Survey, previous studies have mainly used a variable-oriented approach and examined the single dimensions of exhaustion, cynicism and professional efficacy as recommended in the manual [6]. However, we presume that individuals may simultaneously experience shifting intensities across one or more of the three burnout dimensions, and that two or three dimensions is worse than experiencing burnout in only one dimension. Thus viewing burnout as a syndrome, we adopt a person-oriented approach that entails focusing on the co-occurrence of burnout signs on the individual level [14]. Therefore, in addition to studying the single dimensions of burnout, the present study also set out to examine differences between groups of individuals that are defined by their individual configurations of burnout signs.

Teachers work in a complex environment where many different factors may contribute to perceived stress and burnout. Accordingly, we used a broad approach that entailed assessing the psychosocial work environment (e.g. [7, 8]), the physical workload [15], sociodemographic and life-style factors [16], sleep problems [17] and the occurrence of musculoskeletal pain [18, 19]. This approach was informed by our previous experiences and our understanding of the bio-psycho-social-model that underlines that health and disease depend on many factors on different levels and on the interaction between them [20, 21].

In addition, and since male and female teachers perform highly similar work, we also investigate potential gender differences. It is known that women seem to be more affected by stress [22] and emotional exhaustion [23] than males. However, it is often not clear whether these differences are due to the fact that men and women typically have different occupations and/or job positions [24, 25]. Indeed, the gender segregation of the labour market often complicates appropriate comparisons between men and women and risk inducing occupational confounding. Thus, by addressing gender differences among teachers we address the general need for studying men and women in the same occupation [26].

### Aims

The aims of the present study was to examine (a) the occurrence of burnout in a sample of Swedish school teachers, (b) go beyond the traditional view of burnout as a three dimensional construct by creating a combined measure on the individual level reflecting individual configurations of burnout signs, (c) to characterize how burnout was associated with occupational, sociodemographic and life-style factors and (d) to explore any differences between the genders.

## Methods

### Study design and population

The present study, representing the baseline of a planned prospective study, is based on cross-sectional data obtained from a questionnaire about occupational factors, as well as sociodemographic and life-style factors. The questionnaire was directed to 769 teachers (227 men and 542 women) employed at 50 (out of 64 invited) compulsory schools across seven Swedish municipalities. Among the invited schools who declined participation, the most common explanations from the headmasters were “lack of time due to organisational changes and too much administrative work among the teachers”, or “recent change of headmaster/manager”; or “recent involvement in other research projects”. In each school, all teachers educating children in theoretical subjects in school years 4–9 (children aged 10–15 years) were invited. A further inclusion criterion was work at least 50 % of fulltime during the 3 months before completion of the questionnaire.

Out of the 769 teachers 517 (67) responded to the survey [375 women (69), 142 men (63), 357 teachers in school-year 7–9 (68) and 160 teachers in school-year 4–6 (67 %)]. The response-rates in the seven different municipalities ranged from 57 % to 94 %. An additional 27 teachers were excluded due to missing values in the outcome measures, which yielded a total study sample of 490 teachers (64 % of all invited; 356 women and 134 men; Table 1). Only 3 of the study population (4 of the women and 2 % of the men) were daily smokers; smoking was thus not included in the further analysis.

**Table 1:** Occupational, sociodemographic and life-style factors in the total study population (*N* = 490), stratified by gender and year of compulsory school

		All teachers		Females	Males		Year of compulsory school		
Dimensions	Scale	*N*		*N* = 356	*N* = 134	*P* ^*^	Year 4–6 (*N* = 153)	Year 7–9 (*N* = 337)	*P* ^*^
Occupational factors; mean (SD)									
Seniority (years)		490	17 (12)	17 (12)	19 (12)	0.05	19 (13)	17 (12)	0.23
Mechanical exposure index	11–33	481	15.7 (3.4)	15.8 (3.4)	15.4 (3.2)	0.30	15.3 (2.0)	15.9 (4)	0.24
Physical exposure index	7–21	484	10.0 (1.7)	9.9 (1.7)	10.1 (1.8)	0.49	9.6 (1.3)	10.2 (1.8	0.001
Complaints on computer									
workstation arrangements	1–5	482	3.1 (1.1)	3.2 (1.1)	2.9 (1.0)	0.02	2.9 (1.0)	3.2 (1.1)	0.02
Job demands	1–4	486	2.9 (0.4)	2.9 (0.4)	2.8 (0.4)	0.001	2.8 (0.4)	2.9 (0.4)	0.06
Job control (total)	1–4^a^	489	3.2 (0.3)	3.2 (0.3)	3.2 (0.3)	0.08	3.3 (0.3)	3.2 (0.3)	0.01
Decision latitude	1–4^a^	489	3.1 (0.5)	3.1 (0.5)	3.1 (0.5)	0.32	3.1 (0.4)	3.1 (0.5)	0.19
Skill discretion	1–4^a^	489	3.4 (0.3)	3.4 (0.3)	3.2 (0.3)	<0.001	3.4 (0.3)	3.3 (0.3)	0.001
Job support (total)	1–4^a^	489	2.8 (0.4)	2.8 (0.4)	2.8 (0.4)	0.73	2.9 (0.4)	2.8 (0.4)	0.06
Job support management	1–4^a^	489	2.6 (0.6)	2.6 (0.6)	2.6 (0.6)	0.61	2.7 (0.5)	2.6 (0.6)	0.07
Job support colleagues	1–4^a^	487	3.0 (0.4)	3.0 (0.5)	3.0 (0.4)	0.39	3.1 (0.5)	3.0 (0.4)2.8 (0.7)	0.24
Emotional demands	0–4	490	2.8 (0.7)	2.9 (0.7)	2.5 (0.7)	<0.001	2.7 (0.7)	1.7 (0.8)	0.09
Demands of hiding emotions	0–4	490	1.7 (0.8)	1.7 (0.8)	1.6 (0.8)	0.14	1.6 (0.8)	2.4 (0.6)	0.67
Sensory demands	0–4	490	2.4 (0.6)	2.3 (0.6)	2.4 (0.6)	0.34	2.4 (0.6)	2.0 (0.8)	0.80
Leadership	0–4^a^	490	2.0 (0.8)	2.0 (0.8)	2.1 (0.8)	0.50	2.2 (0.7)	4.1 (0.5)	0.01
Self- efficacy	1–5^a^	487	4.1 (0.5)	4.1 (0.5)	4.1 (0.5)	0.33	4.0 (0.5)		0.16
Sociodemographic and life-style factors									
Age, years; mean (SD)		490	48 (11)	47 (11)	48 (11)	0.37	48 (10)	47 (11)	0.30
BMI, points; mean (SD)		473	24 (4)	24 (4)	25 (3)	0.001	24 (4)	24 (4)	0.20
Number of children; mean (SD)		480	1.1 (1.0)	1.1 (1.1)	1.3 (1.2)	0.26	1.2 (1.2)	1.1 (1.1)	0.90
Marital status		480				0.76			0.003
Married/cohabit; n (%)			407 (85)	294 (84)	113 (86)		137 (92)	270 (82)	
Single; n (%)			73 (15)	54 (16)	19 (14)		12 (8.1)	61 (18)	
Neck-shoulder pain; n (%)		484	225 (46)	183 (52)	42 (32)	<0.001	61 (40)	164 (49	0.07
Low-back pain; n (%)		482	162 (34)	129 (37)	33 (25)	0.02	44 (29)	118 (35	0.18
Sleep quality; mean (SD)	1–4^a^	490	2.8 (0.8)	2.7 (0.8)	2.9 (0.8)	0.03	2.9 (0.8	2.8 (0.8	0.12
Personal relaxation time < I hour/day; n (%)		480	106 (22)	83 (24)	23 (18)	0.16	30 (20)	76 (23)	0.49
Domestic work > 21 h/week; n (%)		484	86 (18)	69 (19)	17 (13)	0.11	27 (18)	59 (18)	1.00
Physical exercise occasionally/never; n (%)		485	72 (15)	52 (15)	20 (15)	0.84	14 (9.2)	58 (17)	0.02
Daily smokers; n (%)		486	17 (3.5)	15 (4.2)	2 (1.5)	0.15	4 (2.6)	13 (3.9)	0.48

^(a)^Higher scores among the occupational factors indicate a more unfavourable situation, except where noted

^*^Mann Whitney *U* test of differences between gender and year of compulsory school

### Measures

The questionnaire included questions about burnout, physical and psychosocial working conditions, self-efficacy, sociodemographic and life-style factors, musculoskeletal pain, and sleep quality.

#### Burnout

The Maslach Burnout Inventory-General Survey (MBI-GS [6, 27]) was used to assess burnout. The MBI-GS consists of 16 items rated on a 7-point scale ranging from 0 = “never”; 1 = “a few times a year or less”; 2 = “once a month or less”; 3 = “a few times a month”; 4 = “once a week”, 5 = “a few times a week” and 6 = “every day”. The exhaustion dimension consists of 5 items, the cynicism dimension consists of 5 items, and the professional efficacy dimension consists of 6 items. High scores on exhaustion and cynicism, and low scores on professional efficacy, are indicative of burnout in that specific dimension. In the present study we calculated burnout scores in several ways. To begin with we used the standard approach and thus calculated the mean score for each dimension. However, since the underlying response scale uses non-equidistant scaling, we also made a new type of dichotomous classification that was based on the linguistic meaning of the response scale. Accordingly, for each dimension each item was first dichotomized into 0 = “low” or 1 = “high” by applying a cut-off score of 4 = “once a week”. This level was chosen since it represents a qualitative shift on the non-equidistant ranking scale that the participants respond to, as well as a frequency that begins to be clinically relevant. For the exhaustion and cynicism dimensions, at least three out of five items had to be high in order to be classified as burnout. Regarding the professional efficacy dimension, the subjects were considered to have low professional efficacy if they answered “once a week” or less often in three out of the six items.

To estimate the comorbidity of the burnout dimensions on the individual level we further combined the dichotomized responses into four ordered categories: 0 = subjects reporting low exhaustion, low cynicism and high professional efficacy (referents); 1 = subjects reporting either high exhaustion or high cynicism or low professional efficacy (one out of the three dimensions); 2 = subjects reporting high exhaustion and/or high cynicism and/or low professional efficacy (two out of the three dimensions) and 3 = subjects reporting high exhaustion and high cynicism and low professional efficacy (all three dimensions).

Further, in univariable and multivariable analysis (see Statistical analysis below) the four levels of burnout were reduced to “low” (that is level 0 + 1 combined) and “high” (i.e. level 2 + 3 combined).

#### Physical workings conditions

The mechanical exposure index (MEI [28]) included 11 items of work postures and movements, and the physical exposure index (PHYI [28]) included seven items about physical activity and lifting. In both scales, each item was answered on a three-point scale: 1=”hardly nothing/not at all”, 2 =”somewhat” or 3 =”a great deal”, and the sum scores were used in the analysis (MEI 11–33 possible; PHYI 7–21 possible).

Ergonomic conditions during computer work were assessed by the question “Are you satisfied with the *computer work*-*station arrangements*?” The item was responded to on a five-point scale: 1 = “yes, very satisfied (can work comfortably)”, 2 = “yes, rather satisfied”, 3 = “neither satisfied nor dissatisfied”, 4 = “no, rather dissatisfied”, 5 = “no, very dissatisfied (uncomfortable/strenuous work)”.

#### Psychosocial working conditions

The psychosocial exposure in terms of job demands, job control and job support was measured with a Swedish version of the Job Content Questionnaire (JCQ) [29, 30]. Job demands concerned nine items of e.g. working pace, hard work, excessive demands, time pressure, conflicting demands, and stressful work. Job control concerned nine items of decision latitude (e.g. influence at work, freedom to decide how work should be done) and skill discretion (e.g. development opportunities, skill and creativity). Job support included eight items whereof four items concerned support from management and four items concerned support from co-workers. Responses for each item used a four-point scale, indicating the level of agreement with various statements about conditions at work. The mean value in each dimension was calculated for each individual. Higher numbers indicated higher demands, better control, and better support.

A subset of the Copenhagen Psychosocial Questionnaire [31] was used to measure dimensions defined as emotional demands (three items concerning e.g. emotionally difficult situations and emotional affection by the work), demands on hiding emotions (two items), sensory demands (five items concerning e.g. eye sight, attention, control of body movements and precision), and leadership (eight items concerning planning of work, conflict solving, communication and concern for staff). All questions were answered on a five-point-scale and the mean value in each dimension was calculated for each individual.

#### Self-efficacy

General self-efficacy was assessed with three items [32]. The respondents were asked to decide how well three statements matched themselves: “You can deal with most unexpected events”, “You can solve most problems if you really want to” and “Irrespective of what is going on in your life, you feel that you can handle it”. All items had five response categories: 5 = “always”, 4 = “often”, 3 = “sometimes”, 2 = “seldom”, and 1 = “never/ hardly ever”. The mean score (range 1–5) was used as a continuous predictor. Higher scores indicated greater self-efficacy.

#### Sociodemographic and life-style factors

The participants were asked about *age*, height and weight [*body mass index* (BMI) calculated as kg/height in meter^2^] and *number of children at home*. Further, they were questioned “How much of your leisure time do you normally use for *personal recovery*? (1 = ≥4 h/day; 2 = 3 h/day, 3 = 2 h/day; 4 = 1 h/day; 5 = < 1 h/day and 6 = hardly any time at all); “How many hours a week, do you normally work at home doing cleaning, gardening, cooking, etc.?” (*Domestic work*: 1 = 0–2 h/week; 2 = 3–10 h/week; 3 = 11–20 h/week; 4 = 21–30 h/week and 5 = ≥ 31 h/week), frequency of *physical exercise* (1 = ≥ 5 times/week; 2 = 2–4 times/week; 3 = once a week; 4 = occasionally; 5 = never); *smoking habits* (1 = “have never been smoking”; 2 = “have stopped smoking”; 3 = “smoking, but not daily”; 4 = “smoking daily”).

#### Musculoskeletal pain

The participants were asked about subjective musculoskeletal complaints in the neck-shoulder region and lower back the preceding 12 months and 7 days, following the Nordic Questionnaire [33]. In addition, for each body region, information was collected about the frequency of complaints during the past year using a 5-point scale (never, seldom, sometimes, often, or very often [34] as well as the intensity of complaints on a ten-point-scale, from 0 (none at all) to 10 (very, very severe [35]). A subject was considered to have considerable musculoskeletal pain (subsequently referred to simply as “pain”) if reporting complaints at least “seldom” with an intensity of at least 7 (very severe), or “sometimes” with an intensity of at least 3 (moderate), or “often” or “very often” with an intensity of at least 2 (slight/mild [19]). The condition was defined separately for each body region.

#### Sleep quality

Three items from the Karolinska Sleep Questionnaire [17] were used to assess sleep quality. The items assessed (a) difficulties falling asleep (b) repeated awakening and disturbed/restless sleep, (c) premature awakenings and non-refreshing sleep, were answered on a four-point scale: 1 = “not at all”; 2 =”a little”; 3 = “quite a lot”; 4 = “much”.

### Statistical analysis

The statistical analyses were performed in three steps. In a first step, comparisons of differences between distributions of scores across pairs of independent groups (men/women and teachers in school year 4–6/7–9) were made with the non-parametric Mann Whitney *U* test. In the second step we applied the non-parametric Jonkheere-Terpstra test for trend to examine adverse circumstances in both work and private life across the four ordered levels of increased burnout. In the third and final step the levels of burnout were dichotomized into low (level 0 + 1; reference) and high (level 2 + 3). We used Poisson regression with unit length of follow up for each study participant and robust variance estimation in this step [36]. Prevalence ratios (PRs) and 95 % confidence intervals (CIs) for high levels of burnout were estimated in univariable Poisson regression for all variables (occupational, sociodemographic and lifestyle factors). Further, PRs for high levels of burnout were estimated using multivariable Poisson regression for variables that we considered as relevant risk factors for burn out (irrespective of statistical significance). Thus, neck-shoulder pain, low back pain and sleep quality were excluded since they were considered to be mainly consequences rather than causes of burnout. Mechanical exposure index, physical exposure index, skill discretion, sensory demands, BMI and number of children were considered to be less relevant as risk factors for burnout. Further, age and job support from management were excluded due to a high collinearity (strong correlation) with seniority and leadership, respectively.

The statistical analyses were performed with the IBM SPSS software, version 20 (IBM Corp.). *P*-values ≤ 0.05 were considered as statistically significant.

## Results

### Characteristics of the study population

The women represented a larger fraction of the study population (73 %). The genders reported similar mean scores in several of the physical and psychosocial factors (e.g. in the physical exposure index, job support and leadership; Table 1). However, the women reported higher job demands, higher emotional demands and more complaints on the computer work station arrangements, than the men (Table 1). Further, a larger fraction of the women reported sleep problems and musculoskeletal pain in neck-shoulders and low back, compared to the men. The men had a higher average BMI.

The proportion of women and male teachers differed between the school-years (data not in tables). Thus, 34 of the women and 24 % of the men worked in school-years 4–6 (teaching children aged 10–12 years); while 66 of the women and 76 % of the men worked in school-year 7–9 (children aged 13–15 years).

The teachers in school-year 7–9 reported a higher physical workload and more complaints on the computer work station arrangements than their colleagues in school-year 4–6 (Table 1). Further, they perceived lower job control and worse leadership, than their colleagues who were teaching younger children. A higher fraction of the teachers in school-year 7–9 performed physical exercise occasionally or never.

### Burnout

The women reported a higher mean score in the exhaustion dimension than the men (3.0 vs. 2.6; *p* < 0.01; Table 2), while there was a tendency of a lower mean score in cynicism among the women than in the men (1.6 vs. 1.8; *p* = 0.07). Teachers in school-year 7–9 reported higher cynicism and lower professional efficacy than the teachers in school-year 4–6 (1.8 vs. 1.4; *p* < 0.01, and 4.9 vs. 5.2; *p* < 0.01, respectively).

**Table 2:** MBI exhaustion, cynicism and professional efficacy in the total study population, stratified by gender and year of compulsory school

		All teachers		Females	Males		Year of compulsory school		
Dimensions	Scale	*N*		*n* = 356	*n* = 134	P*	Year 4–6 (*N* = 153)	Year 7–9 (*N* = 337)	P*
Exhaustion; mean (SD)	0–6	490	2.9 (1.4)	3.0 (1.4)	2.6 (1.5)	<0.01	2.8 (1.3)	2.9 (1.5)	0.26
High exhaustion; n (%)			178 (36)	138 (39)	40 (30)	0.07	51 (33)	127 (38)	0.35
Cynicism; mean (SD)	0–6	490	1.6 (1.2)	1.6 (1.2)	1.8 (1.3)	0.07	1.4 (1.0)	1.8 (1.3)	<0.01
High cynicism; n (%)			55 (11)	37 (10)	18 (13)	0.31	11 (7)	44 (13)	0.06
Professional efficacy; mean (SD)	0–6	490	5.0 (0.8)	5.0 (0.8)	5.0 (0.8)	0.65	5.2 (0.7)	4.9 (0.8)	<0.01
Low professional efficacy; n (%)			102 (21)	73 (21)	29 (22)	0.78	19 (12)	83 (25)	<0.01

*Mann Whitney U tests of differences between gender and year of compulsory school

Fifteen percent [73 individuals; 54 women (15) and 19 men (14 %)] of the teachers were classified as having high burnout in at least two out of the three dimensions exhaustion, cynicism and low professional efficacy (i.e. level 2 + 3; Table 3), and 20 (4 %) reported high values in all three dimensions. However, about half of the teachers (51 %) reported low burnout in all the three dimensions (level 0). As expected, there were rising trends of the mean scores in exhaustion and cynicism, and a decreasing trend in professional efficacy, across the four levels of burnout (not in table).

**Table 3:** Occupational, sociodemographic and life-style factors, among 490 teachers (356 females and 134 males), stratified into four levels of burnout; level 0 (no burnout in any dimension), level 1 (burnout in one dimension), level 2 (burnout in two dimensions) and level 3 (burnout in all three dimensions). Further, p-values were presented for univariable models estimating level 0 + 1 *vs*, level 2 + 3

		Level 0	Level 1	Level 2	Level 3	Test for trend	Univariable models level 0 + 1 vs. level 2 + 3
	Scale	(*N* = 248)	(*N* = 169)	(*N* = 53)	(*N* = 20)	*P* ^*^	*P* ^**^
Occupational factors							
Year of compulsory school						0.04	0.003
year 4–6; n (%)		83 (34)	59 (35)	11 (21)	0		
year 7–9; n (%)		165 (66)	110 (65)	42 (79)	20 (100)		
Seniority, years; mean (SD)		17 (13)	19 (12)	15 (10)	13 (8)	0.75	0.007
Mechanical exposure index; mean (SD)	11–33	15.5 (3.3)	15.8 (3.3)	16.1 (3.2)	16.2 (4.2)	0.13	0.24
Physical exposure index; mean (SD)	7–21	9.8 (1.6)	10.1 (1.7)	10.3 (2.2)	10.0 (1.8)	0.07	0.17
Complaints on computer							
workstation arrangements; mean (SD)	1–5	2.9 (1.1)	3.2 (1.0)	3.3 (1.1)	3.9 (1.1)	<0.001	0.003
Job demands; mean (SD)	1–4	2.8 (0.4)	3.0 (0.4)	3.1 (0.4)	3.3 (0.2)	<0.001	<0.001
Job control; mean (SD)	1–4^a^	3.3 (0.3)	3.2 (0.3)	3.1 (0.3)	3.0 (0.3)	<0.001	<0.001
Decision latitude	1–4^a^	3.2 (0.5)	3.0 (0.5)	2.9 (0.4)	2.9 (0.4)	<0.001	<0.001
Skill discretion	1–4^a^	3.4 (0.3)	3.4 (0.3)	3.3 (0.3)	3.1 (0.2)	<0.001	<0.001
Job support; mean (SD)	1–4^a^	2.9 (0.4)	2.8 (0.4)	2.6 (0.5)	2.6 (0.3)	<0.001	<0.001
Job support management	1–4^a^	2.8 (0.5)	2.6 (0.5)	2.2 (0.6)	2.2 (0.5)	<0.001	<0.001
Job support colleagues	1–4^a^	3.1 (0.4)	3.0 (0.4)	2.9 (0.5)	3.0 (0.3)	<0.001	<0.001
Emotional demands; mean (SD)	0–4	2.6 (0.7)	2.9 (0.7)	3.0 (0.6)	3.4 (0.4)	<0.001	<0.001
Demands of hiding emotions; mean (SD)	0–4	1.5 (0.8)	1.8 (0.7)	2.0 (0.8)	2.1 (0.8)	<0.001	<0.001
Sensory demands; mean (SD)	0–4	2.3 (0.6)	2.4 (0.6)	2.4 (0.5)	2.4 (0.6)	0.49	0.84
Leadership; mean (SD)	0–4^a^	2.3 (0.8)	2.0 (0.7)	1.4 (0.8)	1.2 (0.8)	<0.001	<0.001
Self- efficacy; mean (SD)	1–5^a^	4.2 (0.4)	4.0 (0.5)	3.9 (0.4)	3.5 (0.4)	<0.001	<0.001
Sociodemographic and life-style factors							
Gender						0.45	0.78
Men; n (%)		72 (29)	43 (25)	13 (24)	6 (30)		
Women; n (%)		176 (71)	126 (75)	40 (76)	14 (70)		
Age, years; mean (SD)		48 (11)	48 (11)	46 (10)	45 (8.5)	0.31	0.03
BMI, points; mean (SD)		24 (3)	25 (4)	24 (3)	23.5 (3)	0.82	0.50
Number of children; mean (SD)		1.2 (1.2)	1.0 (1.1)	1.2 (1.1)	1.3 (1.1)	0.59	0.42
Marital status						0.60	0.78
Married/cohabit; n (%)		209 (86)	137 (83)	45 (87)	16 (84)		
Single; n (%)		34 (14)	29 (17)	7 (13)	3 (16)		
Neck-shoulder pain; n (%)		97 (39)	86 (52)	29 (56)	13 (65)	0.001	0.03
Low-back pain; n (%)		71 (29)	62 (37)	21 (40)	8 (40)	0.04	0.19
Sleep quality; mean (SD)	1–4^a^	3.1 (0.7)	2.6 (0.9)	2.3 (0.8)	2.4 (0.8)	<0.001	<0.001
Personal relaxation time; mean (SD)	1–6^a^	3.7 (1.4)	3.5 (1.3)	2.9 (1.3)	2.9 (1.1)	0.001	<0.001
Domestic work; mean (SD)	1–5	2.8 (0.9)	2.9 (0.9)	2.8 (0.8)	2.6 (0.5)	0.59	0.22
Physical exercise; mean (SD)	1–5^a^	2.7 (1.1)	2.7 (1.2)	2.6 (1.1)	2.4 (1.1)	0.28	0.21

^*^Jonckheere-Terpstra Test for trend across level 0 – level 3

^**^Univariable Poisson regression: Level 0 + 1 vs. level 2 + 3

^(a)^Higher scores among the occupational factors indicate a more unfavourable situation, except where noted

### Associations between occupational, sociodemographic and life-style factors, and increasing levels of burnout

The physical workload, in terms of the mechanical and physical exposure indices, was not statistically significantly associated with burnout, while teachers with burnout had more complaints on the computer workstation arrangements (*p* <0.001; Table 3).

There was a clear trend in the perception of the psychosocial dimensions across the four levels of burnout (Table 3). While most demand scores (i.e. job demands, emotional demands, demands of hiding emotions) increased with increasing levels of burnout, the job control, job support, leadership and self-efficacy scores decreased (all *p*-values <0.001). Only sensory demands showed no association (*p* = 0.49).

While year of compulsory school was also associated with increasing burnout (i.e. the teachers in school-year 7–9 were more affected; *p* = 0.04), seniority was not (*p* = 0.75).

Although the women had a higher mean score in the dimension exhaustion, there was no association between gender and the rising levels of overall burnout (*p* = 0.45; Table 3). Further, there was no statistically significant difference between the genders in prevalence of high (level 2 + 3) burnout [woman 15 % vs. men 14 %; PR 1.1 (CI 0.7 – 1.7), data not in table].

Musculoskeletal pain (*p* = 0.001) and sleep problems (*p* < 0.001) were associated with a rising level of burnout. Among the life-style factors, only lack of time for personal recovery was statistically significantly associated with burnout (*p* =0.001). There was no significant association between age and burnout (*p* = 0.31).

### Univariable models

As expected, most of the occupational and personal factors that were associated with burnout in the trend-analysis were also statistically significantly associated when using the dichotomized value of burnout in univariable regression models (i.e. level 0 + 1 vs. 2 + 3; Table 3). However, for seniority and age, there was no evident trend across the four levels of burnout (*p* = 0.75 and *p* = 0.31 respectively), but the regression analysis indicated that low seniority and low age were associated with a higher prevalence of level 2 + 3 burnout (*p* = 0.007 and *p* = 0.03, respectively). For low back pain the opposite was true: there was a statistically significant association with burnout in the trend-analysis but no such association in the univariable model.

### Multivariable model

The results indicated that low self-efficacy was clearly associated with the prevalence of level 2 + 3 burnout (*p* < 0.001; Table 4), and the same was true for poor leadership, the perception of high job demands and teaching children in school year 7–9 (*p* = 0.02 – 0.03). Further, there was a tendency that lack of time for personal recovery and low seniority were associated with a higher prevalence (*p* = 0.06 and *p* = 0.08, respectively). There was no statistically significant association between gender and level 2 + 3 burnout (*p* = 0.86).

**Table 4:** Multivariable model in the total study population (*n* = 490) of associations between burnout [level 0 + 1 (= reference) vs. level 2 + 3] and occupational, sociodemographic and life style factors, estimated with Poisson regression, with *p*-values (overall in categorical variables), prevalence ratio (PR) and 95 % confidence intervals (CI)

Dimensions	Scale	*P*	PR (CI 95 %)
Occupational factors			
Year of compulsory school		0.03	
year 4–6			1
year 7–9			2.01 (1.08 – 3.74)
Seniority	years	0.08	0.98 (0.96 – 1.00)
Complaints on computer workstation	1–5	0.70	1.05 (0.83 – 1.32)
Job demands	1–4	0.02	2.28 (1.12 – 4.63)
Decision latitude	1–4	0.42	0.82 (0.51 – 1.33)
Job support from colleagues	1–4	0.16	0.72 (0.46 – 1.13)
Emotional demands	0–4	0.38	1.19 (0.81 – 1.74)
Demands of hiding emotions	0–4	0.13	1.18 (0.95 – 1.47)
Leadership	0–4	0.02	0.69 (0.50 – 0.94)
Self-efficacy	1–5	<0.001	0.37 (0.26 – 0.53)
Sociodemographic and life-style factors			
Gender		0.86	
Men			1
Women			0.96 (0.58 – 1.57)
Marital status		0.28	
Married/cohabitant			1
Single			0.71 (0.38 – 1.32)
Personal relaxation time	1–6	0.06	0.86 (0.74 – 1.01)
Domestic work	1–5	0.17	0.83 (0.64 – 1.08)
Physical exercise	1–5	0.47	1.07 (0.90 – 1.27)

## Discussion

### Principal findings

In the sample of 490 teachers, 15 % had high burnout in at least two out of the three dimensions (i.e. exhaustion, cynicism and low professional efficacy). Four percent of the study population had high burnout in all three dimensions.

Increasing levels of burnout were associated with increasing levels of job demands, emotional demands, and demands of hiding emotions as well as decreasing levels of job control, job support, leadership, and self-efficacy. Likewise, complaints on computer workstation arrangements, musculoskeletal pain, sleep problems and lack of time for personal recovery were all more common with increasing levels of burnout. Teachers in the upper grades were more affected of burnout than teachers in the lower grades. Age was not associated with increasing levels of burnout. The women reported more exhaustion than the men, but there was no association between gender and rising levels of overall burnout, and neither was gender significantly associated with burnout in the multivariable model.

In a multivariable regression analysis, low self-efficacy, poor leadership, high job demands and teaching in higher grades were the indicators most clearly associated with the prevalence of burnout (i.e., level 2 + 3). Thus in the multivariable analysis, it was mainly the combination of working conditions and self-efficacy that was associated with burnout, and not as we presumed the combination of working conditions, sociodemographic and lifestyle factors. Whether those factors that were statistically significant associated in the trend analysis, but not in the multivariable model, have implications for the development of burnout may be further clarified in the follow-up study.

### Strengths and weaknesses of the study

A strength of our study was the fairly novel and person-oriented approach that entailed exploring burnout as a syndrome by studying individual configurations of burnout signs across the three dimensions. While this approach goes against the recommendations in the manual that stipulates that the three dimensions should be measured independently [6] it fits with the general view of how to define a syndrome in psychology (i.e. the co-occurrence of signs and symptoms in relation to one illness or disease [37]). Acknowledging this and that there exist no gold standard for measuring burnout, we believe that there are good reasons for using individual configurations of burnout signs as an analytical unit. Indeed, the response scale in MBI is a frequency scale with non- equidistant steps. Thus, from a mathematical perspective it cannot be assumed to be an interval scale nor depict a linear relationship. Accordingly, calculation of mean scores will introduce a weighting and make the mean score difficult to interpret. However, by embracing the non-equidistant scaling and make the cut-off according to the logic implied by design of the underlying response scale we gain a more robust measure that is easily interpretable. In addition, and in view of clinical experience it makes much sense to start paying increased attention to this exhaustion related symptomatology when people begin to complain on a weekly basis. In extension it seems similarly warranted to focus on the co-occurrence of symptoms under the assumption that more symptoms signal a greater burden to bear. Interestingly, this syndrome approach seems to generate logically coherent and clear results concerning the psychosocial factors.

Another strength was the broad recruitment of schools from seven medium-sized municipalities in southern Sweden. Also the fact that we recruited ordinary schools, making no effort to find neither highly problematic nor extremely prosperous schools, increases the ecological validity. Likewise, our broad approach to the complex school environment that included studying both psychosocial, physical and individual factors in relation to burnout may be regarded as a strength.

Acknowledging this, there are also several limitations of our study, related both to the study design and to the study sample. Regarding the study design, the most obvious limitation is the cross sectional design, which limit causal interpretations between study variables. While not a remedy, this study represent the baseline of a prospective study, and the longitudinal follow up can be expected to bypass some of the inherent limitations. Further, as in all studies based on self-reported exposure and self-reported health, the results must be interpreted with caution. Individuals at risk of burnout may perceive their exposure (physical and psychosocial) to be more demanding than individuals who are doing well, and they therefore may have overestimated their exposure.

Regarding our study sample, both the participation rate and composition of participants needs commenting. The final participation rate of 64 % is acceptable and we could not find any systematic differences in response rates between genders or year of compulsory school. One may, however, speculate that the tendency to respond to the survey may be due to a focus on a relevant issue in the work environment. On the other hand, some individuals may be less prone to respond due to stress and a high workload. The net-effect of these potential explanations is hard to assess. Furthermore, it has been reported that the turnover is high among teachers in Sweden, partly motivated by dissatisfaction with the working conditions [38]. Thus, besides selective participation in the study, there might also be a healthy worker selection that have impacted on the composition of the study sample. For example, teachers with high levels of burnout may have already left the occupation prior to this study. Such a selection would imply lower burnout-scores in the present study. All in all, the above mentioned limitations suggest that the generalizability of the results to all school teachers must be interpreted with some caution.

### The results in relation to other studies

It has earlier been reported that mental disorders and exhaustion were more common in teachers than in non-teachers [39, 40]. Compared to studies using the same measure of Maslach Burnout Inventory, the present study population obtained substantially higher mean scores in exhaustion (mean score 2.9) and cynicism (mean score 1.6), than those in a study of the Finnish general population (mean scores 1.2 and 1.0, respectively [41]). Moreover, the score of exhaustion in the present group exceeded those in a similar study sample of teachers from Finland (mean score 2.1 [2]), while the scores in cynicism were at the same level. Thus, according to the above mentioned studies, our study population seems to be highly affected of exhaustion. Further, the group of individuals with high burnout (i.e. level 2 + 3) seems to be seriously affected, since they obtained mean scores in all three dimensions which were at the same levels as in individuals on long-term sick leave for burnout [42]. However, as regards professional efficacy, the present teachers reported more favourable scores than in comparable study samples [4, 37, 43].

The females reported a higher mean score than the males in the exhaustion-dimension, while the men tended to report higher score than the females in the cynicism-dimension. Similar results were found in the study of Innstrand et al. [5], while Van Horn et al. [1] reported higher scores among male teachers, compared to females, in both dimensions. In the present study, we could not detect any gender difference neither across the rising levels of burnout, nor in the multivariable model. Thus, it doesn’t seem to be any actual gender-difference in the prevalence of severe burnout. However, some of the absence of association between the genders may be explained by the fact that women represented a larger fraction of the study population (73 %), and that the power to detect a difference was fairly low. As our study suggested gender differences in exhaustion, and possibly also in cynicism, one may also speculate that there may be sociocultural variations or differences in individual coping-strategies between the genders. However, further research is needed on this matter.

We observed that self-efficacy, leadership, job demands, year of compulsory school and lack of time for recovery were the factors most clearly associated with burnout. The clear association between low self-efficacy and burnout is in line with the study of Schwarzer and Hallum [10]. However, whether self-efficacy should be regarded as predictor or an outcome may be debated. Similarly, leadership in terms of how the employee perceive the leaders work was also clearly associated with burnout, although the causality could not be assessed. Nevertheless, this association underscores that the relationship between employee and the leader is a potential entrance to dealing with burnout. Not surprisingly was the perception of high job demands also associated with burnout.

Perhaps more interesting is our finding that the teachers in the upper grades were more affected of burnout than those in the lower ones, which is in accordance with the study of Van Horn et al. [1]. There may be several possible explanations. Generally in Sweden, teachers in the lower grades educate only one or a few classes in many subjects, while teachers in higher grades have a few subjects and many classes, and accordingly a higher number of pupils. This may, in turn, increase both the emotional and administrative burden. Further, among younger children, most classes have their own classroom while in higher grades both children and teachers must alter between different facilities. In addition, the interaction with older pupils (teenagers) may be more demanding and the teachers may, to a higher extent, have to deal disciplinary problems and conflicts.

Further the findings showed that lesser time for personal recovery was associated with a rising level of burnout. Recovery prevents strain reactions and health problems from accumulating in the long term [16]. The teachers in the present study were not on sick leave, but previous studies have shown that need for recovery increased sickness absence [44].

It must, however, be emphasized that the negative picture is not clear. In spite of the indications that some individuals may be at risk of severe burnout, about a half of the teachers reported low values in all three dimensions. Thus, as in other occupational groups (e.g. [19]), there was a large inter-individual variation in both the perception of the working conditions, and the response in terms of impaired health. Apart from differences in susceptibility among individuals, and although the teachers theoretically performed the same job, some of the variation may be explained by differences in working conditions between schools (with opportunities of improvements).

### Possible implications

That 15 % of the teachers show signs of burnout in at least two of the three dimensions is worrying, as one could suspect that this increases the risk for sick-leave, job change, early retirement, reduced job performance and, in extension, poorer education of the school-children. Whether this is a consequence of the multiple changes of legislation and educational systems in Sweden, we can only speculate. However, to be observant on similar tendencies may be of interest also in an international perspective.

Due to the high number of teachers at risk of burnout, and to the associations with their perception of occupational and life style factors, a combination of countermeasures on different levels (i.e. societal, organisational and the individual) would be valuable. A combination of countermeasures at the organisational and individual level that improve the leadership and strengthen the teachers’ self-efficacy may have a good effect. Other examples could be improved coordination between various stakeholders, development of the leadership, streamlining of administrative tasks and improved ergonomic conditions during computer work. In addition, and given that lack of time for personal recovery was positively associated with burnout, countermeasures may also include implementing work life balance policies, programmes designed to ease the impact of job demand, and efforts that clarifies the teacher’s responsibilities and norms for good enough work. These countermeasures could probably also decrease the sleep problems.

## Conclusions

The present study adopted a novel scoring approach that on the individual level mapped the co-occurrence of symptoms across the three MBI-GS dimensions of exhaustion, cynicism, and professional efficacy. Utilizing this approach we observed that nearly 50 % of the teachers displayed no signs of burnout whereas 15 % of the teachers displayed signs of burnout in at least two of the three dimensions. Both occupational and sociodemographic factors are associated with burnout levels and men and women have similar prevalence’s of burnout. That circa 15 % of the teachers appear to be in an undesirable psychological state, and that an increasing co-occurrence of burnout signs was associated with several occupational and life-style factors, suggest that countermeasures need to be multifaceted and targeted.

## Abbrevations

BMI: Body mass index
MBI-GS: Maslach burnout inventory – General survay
MEI: Mechanical exposure index
PHYI: Physical exposure index
